# Effect of core materials for core fabrication for dental implants on in-vitro cytocompatibility of MC3T3-E1 cells

**DOI:** 10.1186/s12903-019-0985-0

**Published:** 2019-12-18

**Authors:** Jung-Hyun Park, Hyun Lee, Seen-Young Kang, Junesun Kim, Ji-Hwan Kim

**Affiliations:** 1 Department of Dental Laboratory Science and Engineering, Hana Sciences Hall B #375, Korea University, 145, Anam-ro, Seongbuk-gu, Seoul, 02841 Republic of Korea; 2 Department of Materials Science and Engineering, Hana Sciences Hall B #473, Korea University, 145, Anam-ro, Seongbuk-gu, Seoul, 02841 Republic of Korea; 3 Department of Physical Therapy, College of Health Science, Hana Sciences Hall B #666, Korea University, 145, Anam-dong, Seongbuk-gu, Seoul, 02841 Republic of Korea; 4 Department of Dental Laboratory Science and Engineering, Hana Sciences Hall B #374, Korea University, 145, Anam-ro, Seongbuk-gu, Seoul, 02841 Republic of Korea

**Keywords:** Dental core materials, Pre-osteoblast, Cytocompatibility, Dental prosthetic restoration process

## Abstract

**Background:**

Despite the wide use of dental materials for CAD/CAM system in prosthetic treatment, the effect of the materials, which are used as dental implants core fabricated, on cells involved in dental implant osseointegration is uncertain. This study aimed to investigate and compare the effect of single core materials used for dental implants fabricated by the dental prostheses fabrication process and the CAD/CAM milling method on MC3T3-E1 cells.

**Methods:**

The materials used for prostheses restoration in this experiment were Porcelain Fused Gold (P.F.G), Lithium disilicate glass ceramic (LiSi_2_), Zirconia (ZrO_2_), Nickel-Chromium (Ni-Cr) and Cobalt-Chromium (Co-Cr). MC3T3-E1 cells were cultured and used, the cell adhesion and morphology were observed and analyzed using confocal laser scanning microscopy (CLSM). Methoxyphenyl tetrazolium salt (MTS) and alkaline phosphatase (ALP) assay were used to observe the cell proliferation and differentiation.

**Results:**

CLSM revealed irregular cell adhesion and morphology and the filopodia did not spread in the Ni-Cr specimen group. Significantly high cell proliferation was observed in the ZrO_2_ specimen group. The LiSi_2_ specimen group presented significantly high cell differentiation. Intergroup comparison of cell proliferation and differentiation between the Ni-Cr specimen group and all other specimen groups showed significant differences (*p* < .05).

**Conclusion:**

Cell proliferation and differentiation were observed from the cores, which were fabricated with all specimen groups on cytocompatibility except the Ni-Cr specimen group.

## Background

Studies on the use of biocompatible dental materials for restoration of dental prostheses have been conducted continuously [1]. Studies on the cytocompatibility of biomaterials for dental use include in-vitro and in-vivo experiments; clinical, animal, and cell culture experiments are used to investigate the reactions of biological tissues [2]. Since in-vivo reactions are complex, identifying the part where cells are involved, the steps involved in the reaction, and their outcome is extremely difficult [3]. For the implants used in dentures, surface-treated dental biomaterials that facilitate integration into bones and intraoral tissues are used and lost teeth are replaced considering the tooth shape and oral function of the patient after implantation [4]. While replacing lost teeth, dental materials including metals, ceramics, and composite resins are used for implant prosthetic restorations considering the shape of the lost teeth and oral functions. To achieve the required shape and function after restoration using such artificial materials, functional restorations using these materials must be fabricated. Although the dental restoration materials used in actual clinical practice are based on standards with respect to their physical properties, the potential for risk factors exists due to micro-changes caused during fabrication as the materials undergo a secondary physicochemical fabrication. Nevertheless, studies on the relationship between the negative micro-influence caused in applied restoration and intraoral biological tissues are rare.

Owing to the convenience of dental restorations and the need for biological stability, the demand for implants is gradually increasing [5]. The restorations applied to the implant prosthetic superstructures used most commonly are fabricated entirely in the CAD/CAM system or as a part of restorations that underwent the designing and cutting process [6]. For fabrication of dental restorations, the wax patterns created after the designing process in the CAD/CAM system undergo investment, burn out, casting, polishing or sintering, and pressing. Among dental materials, the materials used to fabricate the dental core of the implant for upper prosthetic restorations are P.F.G, metals including Ni-Cr and Co-Cr, and ceramic materials including Lithium disilicate glass ceramic and zirconia [7–10]. Based on a previous study, among the dental precious metal alloys widely used for prosthetic restorations, gold alloys are known for their excellent cytocompatibility and high corrosion resistance [11]. Although non-precious metal alloys are more utilized due to economic circumstances, chances of metal ions micro-release inside the mouth and various types of corrosion including corrosion, friction, and fatigue due to external compounds are higher than those with gold alloys [12]. Furthermore, complications including inflammation and damage due to implant osseointegration may be caused by the interaction between released non-precious metal ions by the chipping due to corrosion, and other cells and soluble molecules [13].

Release of metal ions from the non-precious metal superstructure after implant placement may influence the intraoral physiological environment and interactions, ultimately causing functional defects [14]. Ni-Cr dental alloys possess relative corrosion resistance when they contain 20% or more Cr; however, corrosive actions are also altered depending on the casting condition, heat treatment, and surface treatment process during the fabrication process of dental restorations [15]. Therefore, restorations of Ni-Cr alloys may alter food taste or cause gingivitis [16]. Ni is one of the metals that causes allergy and triggers local and systemic allergic reactions in sensitive individuals [17]. Therefore, all dental alloys containing Ni should undergo thorough examination before and after clinical application. Considerable endeavors have been made to improve the biological characteristics of superstructures made from non-precious metal alloys that may reduce implant biocompatibility due to the potential release of metal ions [18]. One of the most common recent technologies enhancing the biocompatibility of the non-precious metal alloy superstructure is the use of biocompatible materials such as zirconia and lithium disilicate glass ceramic [19]. Zirconia possesses excellent corrosion resistance and presents in-vivo and in-vitro biocompatibility [20]. It also has great influence on the chemical composition of dental materials and implant adhesive strength [21]. However, studies with cells to investigate implant osseointegration after obtaining the core, which is a superstructure of the implant prepared through the dental restoration fabrication process using such materials, are extremely rare.

Alloys used for dental upper prosthesis consist of 4 or more or 6 or more metals and the composition of these alloys varies. To meet the increasing functional and biological needs for these dental alloy materials, studies on biocompatible materials with respect to the chipping due to stress during the long-term use of the upper prosthesis have been ongoing [22]. Co-Cr and Ni-Cr dental alloys have been widely used for dental prosthetic treatment; however, the biocompatibility has been questioned owing to the metal ions released as these alloys contain the oxidized layer on the surface of the alloys [23]. The P.F.G Gold alloy, which is a gold alloy for dental restoration, is known as the material that does not cause discoloration and oxidation of dental porcelain [24]. Among dental ceramics, Zirconia and Lithium disilicate glass ceramic that are commonly used for the fabrication of dental prostheses can cause failure of Zirconia and Lithium disilicate-based dental restorations since they are vulnerable for the fracture caused by brittle rupture in crown-adhesive material-core structure when excessive stress is loaded on upper structure [25, 26].

**Fig. 1:**
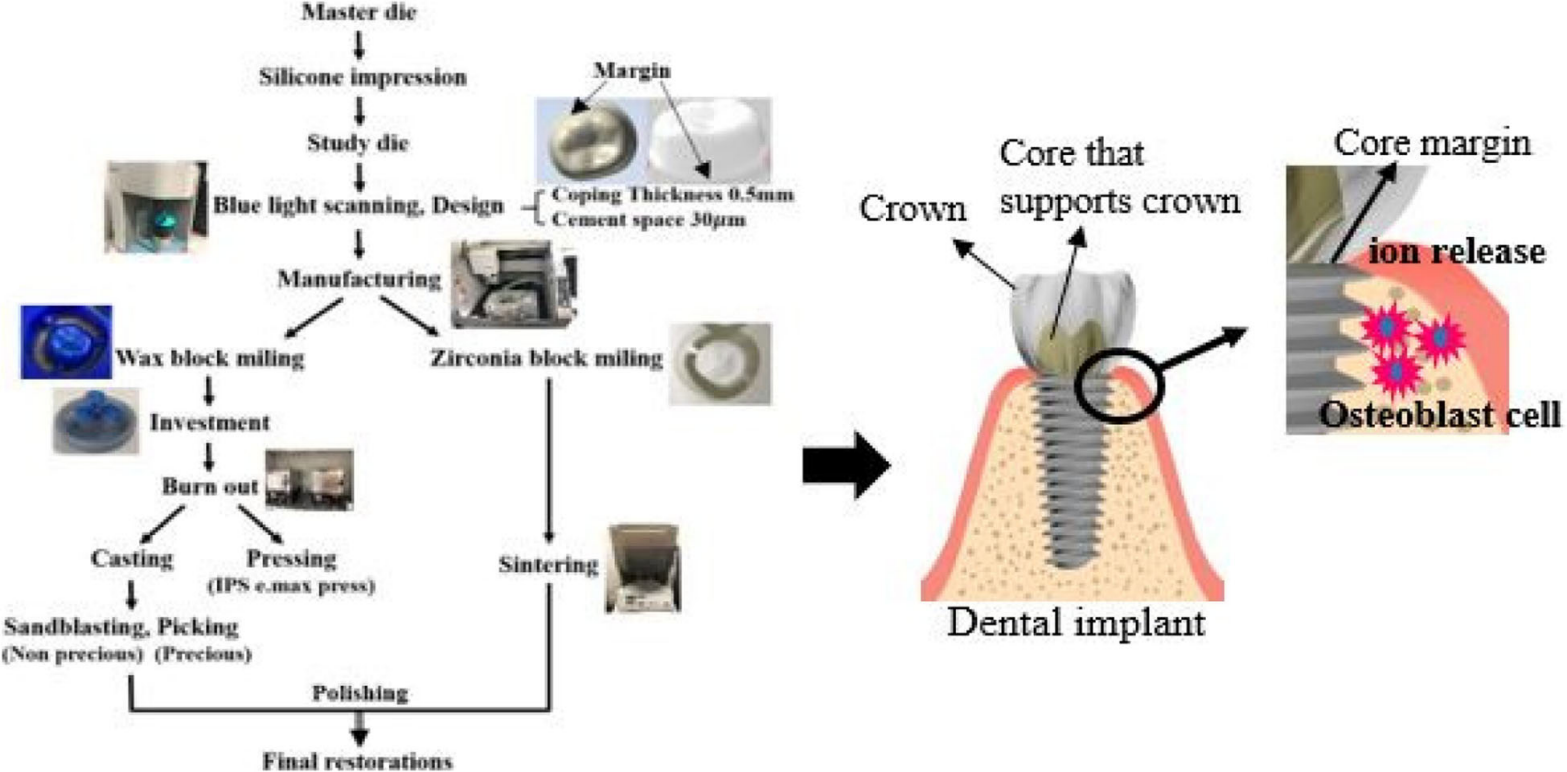
Dental core fabrication process of dental restorations by CAD/CAM milling method and cytocompatibility of the osteoblast cell

In this study, commercially available gold alloys for P.F.G, Co-Cr and Ni-Cr from among the non-precious metal alloys, lithium disilicate glass ceramic that is a ceramic material, and zirconia were used to fabricate single cores that act as superstructures for dental implant prosthetic restorations, through the computer aided design and computer aided manufacturing (CAD/CAM) method (Table 1). The actual manufacturing process was applied according to the manufacturer’s instructions for each material, and after the specimens were prepared, the core for restoring the prosthesis on the implant was prepared. The adhesion, proliferation and differentiation of cells were analyzed using MC3TC-E1 pre-osteoblasts involved in implant osseointegration (Fig. 1).

**Table 1:** Components of materials used to fabricate the dental upper prostheses for the experiment

Dental Materials	Purpose	Composition (wt%)						
P.F.G	Dental Casting	Au	Pt	Others				
		86.2	10.8	3.0				
LiSi_2_	Dental Pressing	SiO_2_	Li_2_O	K_2_O	P_2_O_5_	ZrO_2_	ZnO	Other oxides and ceramic pigments
		57–80	11–19	0–13	0–11	0–8	0–8	0–10
ZrO_2_	Dental Sintering	ZrO_2_ + HfO_2_ + Y_2_O_3_	Y_2_O_3_	HfO_2_	AlO_2_ + Other oxides			
		≥ 99%	> 4.5 - ≤ 6.0	≤ 5	≤ 1.0			
Ni-Cr	Dental Casting	Ni	Cr	Mo	Nb	Al	Si	
		71.85	12.8	9.0	4.0	2.5	0.5	
Co-Cr	Dental Casting	Co	Cr	W	Nb	V	Si	
		59.4	24.5	10.0	2.0	2.0	1.0	

*P.F.G* Myeso X*, LiSi2* IPS e.max press*, ZrO2* ZenostarT*, Ni-Cr* StarLoy C*, and Co-Cr* Verabond 2 V

## Methods

### Preparation of specimens

An acrylic model of the mandibular right first molar (AG-3 ZPVK 36; Frasaco GmbH, Tettnang, Germany) was prepared. A plaster model was fabricated by taking an impression of this model with Silicone rubber (Deguform; Degudent GmbH, Germany) based on the manufacturer’s recommendation. The plaster model was scanned by using a blue light scanner (Identica blue; Medit, Seoul, Korea) and the scanned data were stored in the format of a STL file. The stored STL file was imported to the CAD design program (Exocad; GmbH, Darmstadt, Germany) for designing the single core with 0.5 mm-thickness and 30 μm-cement gap. To fabricate the single core, a wax block (Vipi Block wax, Vipi, Pirassununga, Brazil) and zirconia block (ZenostarT; Wieland Dental GmbH, Pforzheim, Germany) underwent a 5-axis milling process using a milling machine (DWX-50; Roland DG Corporation, Shizuoka, Japan) (Fig. 1).

In this experiment, the wax pattern that was milled for the metal core fabrication underwent the investment, burn out, and casting according to the fabrication process sequence. Using the investment material (Bellavest SH; Bego GmbH, Germany), investment was performed in a certain water/powder ratio according to the manual. Based on the investment material manual for burn out, 1-h holding at 250 °C was done followed by 2-h holding at 900 °C. A high frequency dental casting machine (Dentaurum; Germany) was used for casting the metals including Co-Cr (StarLoy C; DeguDent, Hanau-Wolfgang, Germany) and Ni-Cr (VeraBond 2 V; Aalba Dent, Fairfield, CA, USA). The metal single cores and specimens were sandblasted using a sandblasting machine (Basic quattro IS, Renfert, Germany). The P.F.G (Myeso X, Yesbiogold, Southkorea) was invested using high temperature investment materials (phosphate) and two single cores were fabricated according to the manufacturer’s instructions. An hour after investment, the cores were placed in the burn out furnace and the temperature was increased to 300 °C. After 30 min holding at 300 °C, the temperature was increased to 850 °C and held for another 30 min followed by casting. After the alumina blast, cleansing was performed for an hour in hydrofluoric acid (HF) and another cleansing for HF removal was performed for 10 min using an ultrasonic cleaner. The mixing ratio was 20% of HF diluted in 80% of water in the total volume. To fabricate ceramic cores, the milled zirconia underwent a sintering process using a zirconia sintering machine (Sinterofen H/T Speed, Mihm-Vogt GmbH, Deutsch, Germany) after increasing the temperature to 1650 °C according to the manual of the machine. For lithium disilicate glass ceramic (IPS e.max press, Ivoclar Vivadent Ltd., Germany), a single core and specimen were fabricated by investing, 30-min-setting, and holding the wax pattern, and was milled with Ingot HT shade A2 according to the manual, at 850 °C followed by pressing. After fabrication, all the specimens were polished with P400 SiC paper.

### Cell culture

A pre-osteoblast cell line (MC3T3-E1; ATCC, CRL-2593, Rockville, MD, US) was used in this experiment. MC3T3-E1 cells were cultured at 37 °C in a humidified incubator containing 5% CO_2_. The culture medium used was minimum essential medium (α-MEM: Welgene Co., Ltd., Seoul, Korea) containing 10% fetal bovine serum (FBS), 1% penicillin streptomycin, 10 mM β-glycerophosphate (Sigma), and 10 μg/mL ascorbic acid. Cell culture maintenance was performed by washing the cells with Dulbecco’s phosphate-buffered saline (DPBS) followed by cell detachment using trypsin-EDTA. The detached cells were then suspended in culture medium, centrifuged, counted using trypan blue dye, plated in culture plates (10 mL, 3 × 10^4^ cells/mL), and cultured at 37 °C.

### Cell attachment analysis

**Fig. 2:**
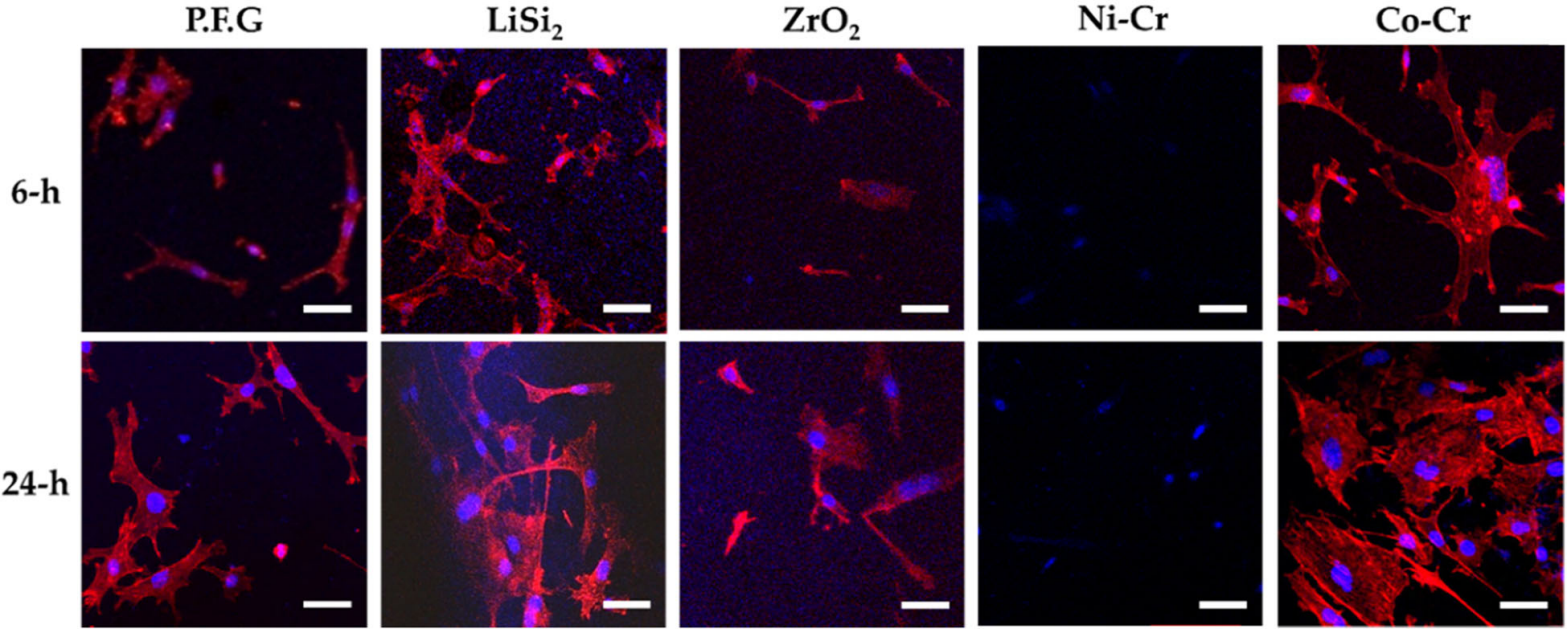
Outcome of measurement in the marginal region of P.F.G, LiSi_2_, ZrO_2_, Ni-Cr, and Co-Cr using CLSM after 6-h and 24-h culture of MC3T3-E1 cells (blue: nuclei, red: cytoplasm, and scale bar: 50 μm)

A piece of size 10 × 10 mm, which is suitable for cell culture, was prepared from the marginal region of each of the metal and ceramic cores of the five completed specimens and the surface and edge of the specimens were trimmed in the shape of a plate. To prepare for confocal laser scanning microscopy (CLSM), pre-osteoblast MC3T3-E1 (3 × 10^4^ cells/mL) cells were cultured for 6 h and 24 h on each of the specimens sterilized with 70% ethanol (Fig. 2). The specimens were then fixed with 4% paraformaldehyde for 10 min, permeabilized with 0.1% Triton X, and blocked with 1% bovine serum albumin. The specimens were then incubated with phalloidin and 4′,6-diamidino-2-phenylindole to stain the cellular actin and nuclei, respectively. Cell morphology was compared using CLSM (C1 Plus; Inverted IX81, Olympus, Japan) (Fig. 2).

### Analysis of cell proliferation

Four plates of 10 × 10 × 3 mm for each of the metal and ceramic specimens were prepared and underwent the CAD/CAM method of fabrication and the fabrication of dental restorations. The plates were then placed into the wells and underwent the methoxyphenyl tetrazolium salt (MTS) assay with 3-(4,5-dimethylthiazol-2-yl)-5-(3-carboxymethoxyphenyl)-2-(4-sulfophenyl)-2H-tetrazolium (MTS, Promega, Madison, US) assay. After placing the cells on each of the four specimens, the medium was removed after 5 days of incubation, which is the observation period, and the specimen was cleansed with DPBS. After mixing 100 μl of MTS per mL of FBS-containing medium, the solution was added to each of the specimens and incubated at 37 °C for 2 h. Next, 200 μl of the medium was placed into a 96-well and absorbance was measured at 490 nm using a Micro-reader (Model 550; BioRad, USA).

### Analysis of cell differentiation

In this experiment, proteins on the specimens were extracted and ALP activity assay was performed by measuring alkaline phosphatase (ALP) in the same amount of protein. For the ALP activity assay, 10 mM β-glycerophosphate (β-GP) and 50 μg/mL ascorbic acid (AA) were added to the medium. Four plates of 20 × 20 × 3 mm for each of the metal and ceramic specimens prepared through the CAD/CAM method and dental restorations fabrication process, and were cultured for 14 days. The culture medium was changed to medium containing β-GP (10 mM) and ascorbic acid (50 mg/mL), and was replaced every 3 days. After cleansing with DPBS, cells were detached with 4 mL of Trypsin-EDTA, the suspension was centrifuged, and the supernatant was removed. Using the protein solution and Triton X-100, the final volume of the sample was adjusted to 100 μl based on normalization to protein production obtained by protein assay. Next, 50 μl of p-Nitrophenyl phosphate (pNPP) (Sigma, USA), the matrix solution, was added and incubated at 37 °C for 1 h. ALP activity was evaluated by measuring the absorbance at 405 nm using a UV-vis spectrometer (Victor 3, Perkin Elmer, USA).

### Statistical analysis

Kolmogorov-Smirnov test and Shapiro-Wilk test were performed for testing normality and Levene’s test was performed for homogeneity of variance. After performing Mann-Whitney U-test based on non-parametric statistics, statistically significant differences were determined using Bonferroni’s post-hoc test. Tukey’s post-hoc test was performed for the post hoc after one way-ANOVA. Intergroup comparative analysis was done at the 95% confidence level. Statistical significance was presented as *p < .05, **p < .01, and ***p < .001. IBM SPSS (IBM SPSS 25.0; Inc., Chicago, IL, USA) was used for statistical analysis.

## Result

### Cell attachment analysis

In the in-vitro cell experiment for specimens prepared by dental prosthetic restorations, the adhesiveness of MC3T3-E1 cells was presented in 3-dimensional images (Fig. 2). Multiple nuclei (stained in blue) and spread cytoplasm (stained in red) are presented, respectively.

Measurement using CLSM after 6 h and 24 h cell culture revealed sufficient cytocompatibility of P.F.G, Lithium disilicate glass ceramic, Zirconia, and Co-Cr (Fig. 2) as the cellular actin and nuclei were adhered together and actively spread out. Among the metal alloys, Co-Cr presented the highest cell-adhesive distribution whereas Lithium disilicate glass ceramic among the ceramic materials presented the highest cell-adhesive distribution. Although cell-adhesive distribution was observed with P.F.G, Lithium disilicate glass ceramic, Zirconia, and Co-Cr, MC3T3-E1 cells were not able to spread out on Ni-Cr.

### Analysis of cell proliferation

**Fig. 3:**
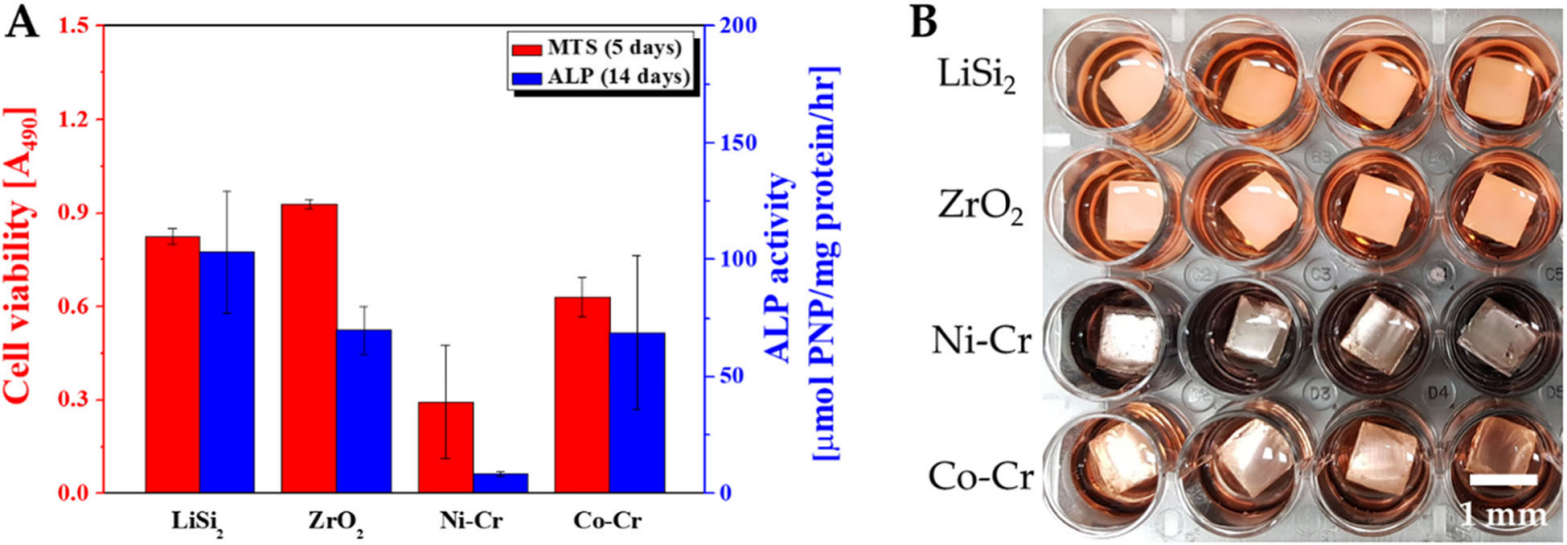
The methoxyphenyl tetrazolium salt (MTS) and alkaline phosphatase (ALP) assay absorbance difference. **a** Absorbance of MTS and ALP in MC3T3-E1 cells. **b** Reaction with indicators due to the release of Ni-Cr metal ions during cell culture

The proliferation of MC3T3-E1 cells in vitro was measured using MTS assay at 5 days after cell proliferation. The measurement presented intergroup absorbance differences between the metal specimens and ceramic specimens prepared through the dental restoration fabrication process with a statistical significance level of 0.05 at 95% confidence level (Table 2). Since the mean and standard deviation of the measurement in the Ni-Cr group after 5 days was 0.3 ± 0.2, significantly smaller values were obtained compared to other groups, whereas a significantly high value was obtained from the zirconia group with the mean and standard deviation at 0.9 ± 0 (Fig. 3a). Zirconia was found to induce more cell proliferation (Fig. 3a). Cell proliferation was higher in Lithium disilicate glass ceramic, Zirconia, and Co-Cr compared to Ni-Cr. In contrast, Ni-Cr presented reduced cell proliferation (Fig. 3a). Intergroup comparison of cell proliferation (Table 2) showed statistically significant differences between Ni-Cr and Lithium disilicate glass ceramic, between Ni-Cr, and zirconia (*p* < .001), between Co-Cr and Ni-Cr (*p* < .01), and between zirconia and Co-Cr (*p* < .01) (Table 2). However, there was no statistically significant difference between Lithium disilicate glass ceramic and Zirconia and between Lithium disilicate glass ceramic and Co-Cr (*p* > .05) (Table 2).

**Table 2:** Intergroup statistical comparisons of methoxyphenyl tetrazolium salt (MTS)

					95% C.I	
Group	Mean	SD	Median	*P*-value	lower	upper
LiSi_2_-ZrO_2_	.877	.059	.880	.934	−.322	.113
LiSi_2_-NiCr	.559	.308	.638	.000^***^	.312	.748
LiSi_2_-CoCr	.727	.113	.742	.094	−.023	.412
ZrO_2_-NiCr	.611	.360	.700	.000^***^	.417	.853
ZrO_2_-CoCr	.779	.165	.804	.006^**^	.081	.517
CoCr-NiCr	.462	.219	.527	.002^**^	.118	.554

* *p* < .05, ** *p* < .01, *** *p* < .001

### Analysis of cell differentiation

Figure 3 shows the outcomes of MC3T3-E1 cell differentiation on metal and ceramic specimens measured through the ALP assay after 14 days of culture (Fig. 3). The mean and standard deviation of the measurement in Lithium disilicate glass ceramic was significantly high as 103 ± 26, whereas that in Ni-Cr was significantly low as 8 ± 1 (Fig. 3a). Intergroup comparison revealed statistically significant differences between Zirconia and Ni-Cr, and between Co-Cr and Ni-Cr (*p* < .05) with a statistically significant level of 0.05 at 95% confidence (Table 3). The difference between the Lithium disilicate glass ceramic group and the Ni-Cr group was statistically significant (*p* < .01) (Table 3). Since the *p*-value presented between Ni-Cr group and all other groups was *p* < 0.05, the intergroup difference was statistically significant (Table 3). However, intergroup comparison among Lithium disilicate glass ceramic, Zirconia, and Co-Cr did not present statistically significant differences (Table 3). Lithium disilicate glass ceramic presented high ALP activity, whereas Ni-Cr showed low ALP activity (Fig. 3a). Compared to Ni-Cr, cell differentiation was more active in Lithium disilicate glass ceramic, zirconia, and Co-Cr (Fig. 3a).

**Table 3:** Intergroup statistical comparison of alkaline phosphatase (ALP) assay

					95% C.I	
Group	Mean	SD	Median	*P*-value	lower	upper
LiSi_2_-ZrO_2_	86.637	25.464	78.663	.301	−23.050	90.055
LiSi_2_-NiCr	55.871	54.594	42.875	.003^**^	38.481	151.587
LiSi_2_-CoCr	86.092	32.645	88.011	.278	−21.960	91.145
ZrO_2_-NiCr	39.120	34.316	35.606	.034^*^	4.979	118.085
ZrO_2_-CoCr	69.341	21.857	69.608	1.000	−55.462	57.643
CoCr-NiCr	38.575	39.149	21.884	.037^*^	3.889	116.994

* *p* < .05, ** *p* < .01

## Discussion

In this study, dental cores were fabricated with materials that are used for implant superstructures, through the dental restorations fabrication process, Cell adhesion, morphology, proliferation, and differentiation of MC3T3-E1 pre-osteoblast cells involved in implant osseointegration, on each core were analyzed, and implant cytocompatibility was investigated. In most previous studies on implants, cytocompatibility has been studied through in-vitro cell experiments using osteoblast cells and titanium or titanium alloys [27]. However, there have been few studies on osteoblast cells and the core, which is the superstructure of the implant prepared through the actual process of dental restoration fabrication.

The fabrication process of the dental restorations is an important process requiring professional technique and proficiency, and appropriate dental materials as the process is for the restoration of lost teeth while restoring the aesthetic aspect desired by the patient and the functional intraoral and dental aspects [28]. In this experiment, the cores that form the superstructure of the implant were fabricated using materials including P.F.G, which is a gold alloy, Co-Cr alloy and Ni-Cr alloy, which are non-precious metal alloys, and Lithium disilicate glass ceramic and zirconia, which are ceramic materials. Other than precious metals, non-precious metals, ceramic, various biocompatible poly-substances such as polymers and hybrid-resin ceramic are available [29]. Use of such biocompatible dental materials can enhance the patient’s satisfaction on the restorations in the aesthetic and functional aspects [30].

During dental restorations fabrication, sandblasting surface treatment plays an important role for metal materials in removing the impurities and enhancing the bonding strength between the core and metal materials [31]. In case of ceramic materials, sintering plays an important role of enhancing the intermolecular bonding strength [32, 33].

In this study, the cytocompatibility of the specimen fabricated through dental CAD/CAM restorations fabrication procedure was investigated through in-vitro experiments using MC3T3-E1 cells, which are pre-osteoblast cells involved in implant osseointegration. The core was prepared at the step just before the oral application of the patients, observation of cell adhesion in margin area was possible. As shown below, margin area of core is the part where chipping or fracture occur after long period of use. However, since the margin area of core is manufactured in a curved form, it cannot be utilized for in vitro tests which requires consistent dimension. Thus, form of a plate was made in order to conduct in vitro experiments.

For in vitro assessments, culturing time for each tests were set considering the stability and degree of proliferation. To obtain stable and time dependent initial attachment of cells on the specimens, 6 h and 24 h were chosen, and early stage of proliferation was confirmed for 5 days. Characterization of degree of differentiation, 14 days were chosen since it is suitable to avoid overgrowth of cells which could lead to apoptosis and to gather sufficient amount of ALP for comparison. Cell culture was performed under aseptic conditions. The experimental procedure using CLSM includes a post-treatment process and has disadvantages of difficulties in cell adhesion due to errors arising during the experimental procedure or environmental factors when proficient skills are not mastered.

In this experiment, P.F.G was used for CLSM measurement and the surface and component analysis. CLSM measurement of P.F.G showed proper cell adhesion and an active spread of MC3T3-E1 cells (Fig. 2). This is consistent with previous studies suggesting sufficient biocompatibility, corrosiveness, and corrosion resistance of the dental gold alloys; hence, the prostheses fabricated of the dental gold alloys among other metal alloys were biocompatible [34, 35]. However, this experiment showed more cell adhesive distribution on Co-Cr among the materials used for dental cores compared to P.F.G in both of 6 h and 24 h of culturing. Cell morphology and adhesion observed by CLSM measurement showed that the cells could not spread and proliferate on the core made of Ni-Cr, and the non-precious metal alloys in particular. In general, studies on the intraoral tissue irritation caused by Ni-Cr alloys have been of the interest and nickel cytotoxicity including allergic reactions have been a real problem [36].

In the experiment on cell proliferation and differentiation, Co-Cr, Ni-Cr, zirconia, and lithium disilicate glass ceramic underwent the dental restoration fabrication procedure (Fig. 1) to form of 10 × 10 × 3 mm and 20 × 20 × 3 mm, according to the sizes of the cell culture plate. From the MTS assay for cell proliferation and ALP assay for cell differentiation, a color change of the indicator to blue was observed due to reaction with the ions released during cell culture (Fig. 3b). According to previous studies, Lithium disilicate and zirconia are known to have no pH change after immersion [37], and Co-Cr is also known to be not dissolution in the oral cavity. Therefore, it is considered that it does not cause internal environmental changes [18, 38]. On the other hand, it seems that there is a change in pH of the Ni-Cr alloy [39], and the change in pH affects cytotoxicity. Also, the restorations fabricated from Ni-Cr alloys influenced the tissue cells surrounding the implanted teeth, causing side effects that may result in cytotoxicity and allergy, and interrupted the metabolism of cytokines and cells that play pivotal roles in the inflammatory process due to the release of metal ions [40]. On the other hand, zirconia has been known for its advantage of lowering the risk of inflammatory reaction in tissues adjacent to the implant by reducing the adhesive strength and biofilm accumulation of the bacteria [41].

Therefore, the in-vitro experiment performed in this study is valuable as understanding and recognition of the characteristics and related cytocompatibility of materials used for dental restorations is essential for the appropriate choice and use of dental materials during actual treatment procedures [42]. The success or failure of various dental restoration treatments using dental materials is determined by the appropriate choice and accurate handling of the dental materials possessing suitable characteristics [43]. This is ultimately linked to cytocompatibility that the dental materials react with the patient’s intraoral tissues [44]. In the implant structure consisting of fixtures, abutments and cores, experiments were carried out under the assumption that chipping or breaking of the cores after a long period of use causes micro-leakage and may be involved in implant osseointegration. The core margin used for the superstructure of the implant touches the teeth and gums. However, after placement of the implant, micro-leakage of ions can occur from the marginal region of the core due to friction inside the mouth or long-term use [45, 46].

This study is related to the in-vitro cell compatibility study on the effect of dental core on osteoblasts, but it is necessary to analyze the stability, reaction and effectiveness of dental materials and tissues in the oral cavity more accurately. In-vivo experiments are difficult to perform routinely because they require more time or method than in-vitro experiments. However, the bio-reactivity between the dental material and the oral tissue can be directly observed, and the result of the analysis is considered to be a reliable biocompatibility evaluation.

Hence, in-vivo study through clinical experiments and animal experiments to confirm the biocompatibility of dental materials are thought to be required. With the recent development of 3D printing technology, various experiments using MC3T3-E1 pre-osteoblast cells involved in osseointegration, are expected by studying novel materials and novel fabrication methods to seek for biocompatible dental materials.

In this study, MC3T3-E1 pre-osteoblast cells involved in implant osseointegration were used to analyze the cytocompatibility characteristics including cell adhesion, morphology, proliferation, and differentiation of gold alloy, non-precious metal alloy, and ceramic cores fabricated through the CAD/CAM milling method and the dental restorations fabrication process. MTS assay conducted for the comparison of cell proliferation revealed reduced cell proliferation in the Ni-Cr specimen at 5 days after cell culture. ALP assay for cell differentiation showed that the Ni-Cr specimen had the lowest cell activity. All other specimens presented more uniform cell adhesive distribution and more active cell proliferation and differentiation compared with Ni-Cr. Additionally, cell adhesion, proliferation, and differentiation were more active in dental ceramic materials than in metal materials. However, Co-Cr was found to be similar to the ceramic material because there was no significant difference from the ceramic material.

## Conclusions

The conclusions of this study are as follows: P.F.G, Co-Cr, Lithium disilicate glass ceramic, and zirconia dental cores presented more active cell adhesive distribution compared with Ni-Cr core. Cytocompatibility for implant was confirmed in lithium disilicate glass ceramic, zirconia, P.F.G, Co-Cr. This indicates that the use of lithium disilicate glass ceramic, zirconia, P.F.G, or Co-Cr is desirable when fabricating the core, which is the superstructure of the implant, during the fabrication process of dental restorations.

## Data Availability

The datasets used and/or analysed during the current study are available from the corresponding author on reasonable request.

## Abbreviations

ALP: Alkaline phosphatase assay
CAD/CAM: Computer aided design/Computer aided manufacturing
CLSM: Confocal laser scanning microscopy
Co-Cr: Cobalt-Chromium
LiSi_2_: Lithium disilicate glass ceramic
MTS: Methoxyphenyl tetrazolium salt
Ni-Cr: Nickel-Chromium
P.F.G: Porcelain Fused Gold
ZrO_2_: Zirconia

## References

[1] Caldas IP, Alves GG, Barbosa IB, Scelza P, de Noronha F, Scelza MZ (2018). In vitro cytotoxicity of dental adhesives: a systematic review. Dent Mater.

[2] Bevilacqua L, Milan A, Del Lupo V, Maglione M, Dolzani L (2018). Biofilms developed on dental implant titanium surfaces with different roughness: comparison between in vitro and in vivo studies. Curr Microbiol.

[3] Shahi S, Özcan M, Maleki Dizaj S, Sharifi S, Al-Haj Husain N, Eftekhari A, Ahmadian E (2019). A review on potential toxicity of dental material and screening their biocompatibility. Toxicol Mech Methods.

[4] Toti P, Marconcini S, Enrica G, Pedretti G, Barone A, Covani U (2018). The influence of prosthesis design on the outcomes of tooth implants immediately placed and loaded by means of one-piece titanium machined restoration. J Oral Implantol.

[5] Beikler T, Flemmig TF (2015). EAO consensus conference: economic evaluation of implant-supported prostheses. Clin Oral Implants Res.

[6] Kapos T, Evans C (2014). CAD/CAM technology for implant abutments, crowns, and superstructures. Int J Oral Maxillofac Implants.

[7] Bayne SC, Ferracane JL, Marshall GW, Marshall SJ, van Noort R (2019). The evolution of dental materials over the past century: silver and gold to tooth color and beyond. J Dent Res.

[8] Bilgin MS, Erdem A, Dilber E, Ersoy İ (2016). Comparison of fracture resistance between cast, CAD/CAM milling, and direct metal laser sintering metal post systems. J Prosthodont Res.

[9] Jian Y, Dao L, Wang X, Zhang X, Swain MV, Zhao K (2019). Influence of veneer pore defects on fracture behavior of bilayered lithium disilicate glass-ceramic crowns. Dent Mater.

[10] Kunz P, Fernandes ABF, Cunha LF, Correr GM, Gonzaga CC (2018). Influence of veneering technique on the fit of metal-ceramic crowns. Dent Mater.

[11] Knosp H, Holliday RJ, Corti CW (2003). Gold in dentistry: alloys, uses and performance. Gold Bull.

[12] Nierlich J, Papageorgiou SN, Bourauel C, Hültenschmidt R, Bayer S, Stark H, Keilig L (2016). Corrosion behavior of dental alloys used for retention elements in prosthodontics. Eur J Oral Sci.

[13] Wataha JC, Schmalz G (2009). Dental alloys. In Biocompatibility of Dental Materials.

[14] Upadhyay D, Panchal MA, Dubey RS, Srivastava VK (2006). Corrosion of alloys used in dentistry: a review. Mater Sci Eng A.

[15] Liliana P, Elena SC, Virgil CL, Laurentiu DM, Daniel PS (2018). Corrosion behavior of Ni-Cr dental casting alloys. Int J Electrochem.

[16] Geurtsen W (2002). Biocompatibility of dental casting alloys. Crit Rev Oral Biol Med.

[17] Lu Y, Chen W, Ke W, Wu S (2009). Nickel-based (Ni–Cr and Ni–Cr–be) alloys used in dental restorations may be a potential cause for immune-mediated hypersensitivity. Med Hypotheses.

[18] Oyar P, Can G, Atakol O (2014). Effects of environment on the release of Ni, Cr, Fe, and co from new and recast Ni-Cr alloy. J Prosthet Dent.

[19] Elsaka SE, Elnaghy AM (2016). Mechanical properties of zirconia reinforced lithium silicate glass-ceramic. Dent Mater.

[20] Takaba M, Tanaka S, Ishiura Y, Baba K (2013). Implant-supported fixed dental prostheses with CAD/CAM-fabricated porcelain crown and zirconia-based framework. J Prosthodont.

[21] Pieralli S, Kohal RJ, Hernandez EL, Doerken S, Spies BC (2018). Osseointegration of zirconia dental implants in animal investigations: a systematic review and meta-analysis. Dent Mater.

[22] Wataha JC (2000). Biocompatibility of dental casting alloys: a review. J Prosthet Dent.

[23] Ming PP, Shao SY, Qiu J, Yu YJ, Chen JX, Yang J, Tang CB (2017). Corrosion behavior and cytocompatibility of a co–Cr and two Ni–Cr dental alloys before and after the pretreatment with a biological saline solution. RSC Adv.

[24] Tsai MH. U.S. patent no. 4,194,907. 1980; Washington, DC: US Patent and Trademark Office.

[25] Su N, Yue L, Liao Y, Liu W, Zhang H, Li X, Shen J (2015). The effect of various sandblasting conditions on surface changes of dental zirconia and shear bond strength between zirconia core and indirect composite resin. J Adv prosthodont.

[26] Souza MT, Peñarrieta-Juanito GM, Henriques B, Silva FS, de Oliveira APN, Souza JC (2018). Lithium-zirconium silicate glass-ceramics for restorative dentistry: physicochemical analysis and biological response in contact with human osteoblast. Materialia.

[27] Hotchkiss KM, Sowers KT, Olivares-Navarrete R (2019). Novel in vitro comparative model of osteogenic and inflammatory cell response to dental implants. Dent Mater.

[28] Miyazaki T, Hotta Y, Kunii J, Kuriyama S, Tamaki Y (2009). A review of dental CAD/CAM: current status and future perspectives from 20 years of experience. Dent Mater J.

[29] Bajraktarova-Valjakova E, Korunoska-Stevkovska V, Kapusevska B, Gigovski N, Bajraktarova-Misevska C, Grozdanov A (2018). Contemporary dental ceramic materials, a review: chemical composition, physical and mechanical properties, indications for use. Open access Maced J Med Sci.

[30] Guess PC, Schultheis S, Bonfante EA, Coelho PG, Ferencz JL, Silva NR (2011). All-ceramic systems: laboratory and clinical performance. Dent Clin.

[31] Yun JY, Ha SR, Lee JB, Kim SH (2010). Effect of sandblasting and various metal primers on the shear bond strength of resin cement to Y-TZP ceramic. Dent Mater.

[32] Denry I, Kelly JR (2014). Emerging ceramic-based materials for dentistry. J Dent Re.

[33] Hallmann L, Ulmer P, Kern M (2018). Effect of microstructure on the mechanical properties of lithium disilicate glass-ceramics. J Mech Behav Biomed Mater.

[34] Manaranche C, Hornberger H (2005). Corrosion and biocompatibility of dental alloys. Eur Cell Mater.

[35] Demann ET, Stein PS, Haubenreich JE (2005). Gold as an implant in medicine and dentistry. J Long-Term Eff Med Implants.

[36] Alp G, Çakmak G, Sert M, Burgaz Y (2018). Corrosion potential in artificial saliva and possible genotoxic and cytotoxic damage in buccal epithelial cells of patients who underwent Ni-Cr based porcelain-fused-to-metal fixed dental prostheses. Mutat Res Genet Toxicol Environ Mutagen.

[37] Forster A, Ungvári K, Györgyey Á, Kukovecz Á, Turzó K, Nagy K (2014). Human epithelial tissue culture study on restorative materials. J Dent.

[38] Hancu V, Comaneanu RM, Coman C, Filipescu AG, Ghergic DL, Cotrut MC (2014). In vitro studies regarding the corrosion resistance of NiCr and CoCr types dental alloys. Rev Chim (Bucharest).

[39] Bojinov M, Fabricius G, Kinnunen P, Laitinen T, Mäkelä K, Saario T, Sundholm G (2000). The mechanism of transpassive dissolution of Ni–Cr alloys in sulphate solutions. Electrochim Acta.

[40] Schmalz G, Garhammer P (2002). Biological interactions of dental cast alloys with oral tissues. Dent Mater.

[41] Schünemann FH, Galárraga-Vinueza ME, Magini R, Fredel M, Silva F, Souza JC, Henriques B (2019). Zirconia surface modifications for implant dentistry. Mater Sci Eng C.

[42] Anusavice KJ, Shen C, Rawls HR (2014). Phillips' science of dental materials-E-book. Elsevier.

[43] Anusavice KJ (2012). Standardizing failure, success, and survival decisions in clinical studies of ceramic and metal–ceramic fixed dental prostheses. Dent Mater.

[44] Hasnain MS, Ahmad SA, Chaudhary N, Minhaj MA, Nayak AK (2019). Degradation and failure of dental composite materials. In Applications of Nanocomposite Materials in Dentistry.

[45] Mutlu-Sagesen L, Ergun G, Karabulut E (2011). Ion release from metal-ceramic alloys in three different media. Dent Mater J.

[46] Sailer I, Balmer M, Husler J, CHF H, Kanel S, Thoma DS (2018). 10-year randomized trial (RCT) of zirconia-ceramic and metal-ceramic fixed dental prostheses. J Dent.

